# The economic burden of respiratory syncytial virus and other acute respiratory infections among children <2 years old seeking care at a tertiary facility in Accra, Ghana

**DOI:** 10.7189/jogh.16.04040

**Published:** 2026-05-29

**Authors:** Farina Leonie Shaaban, Joycelyn Dame, An Nguyen, Neele Rave, Clint Pecenka, Louis J Bont, RSV GOLD III – Health Economics Study Group, Rosemary Akuaku, Louis J Bont, Joycelyn Dame, Amma Ekem, Bamenla Goka, An Nguyen, Ebenezer Ntow, Kwabena A Osman, Clint Pecenka, Neele Rave, Farina L Shaaban

**Affiliations:** 1 Department of Paediatrics, University Medical Centre Utrecht, Utrecht, the Netherlands; 2 Department of Child Health, University of Ghana Medical School, College of Health Sciences, Accra,; 3 Center for Vaccine Innovation and Access, PATH, Ho Chi Minh City, Vietnam; 4 Center for Vaccine Innovation and Access, PATH, Seattle, Washington, USA

## Abstract

**Background:**

The global burden of the respiratory syncytial virus (RSV) disproportionately affects low- and middle-income countries (LMICs). The World Health Organization’s Strategic Advisory Group of Experts on Immunisation recently recommended the global implementation of emerging RSV immunisation strategies to protect infants. Cost of illness data are needed to estimate the economic burden of RSV and evaluate the introduction of these immunisation strategies in LMICs. Here, we estimated RSV-associated costs in Ghana.

**Methods:**

We measured resource utilisation from the societal, health system, and household perspectives for children <2 years old receiving care for (severe) acute respiratory infections at Africa’s third largest referral centre located in Accra, Ghana. During the 2023 respiratory season, children were tested for RSV using molecular point-of-care testing at inpatient and outpatient facilities. Direct medical, direct non-medical, and indirect cost data were collected *via* questionnaire-based caregiver interviews and gathered from hospital records. Interviews took place at the index visit, upon discharge, and during follow-up (four–six weeks post discharge).

**Results:**

Of 128 participating children, 59 (46%) and 69 (54%) received care at outpatient and inpatient facilities, respectively. RSV was detected in 58 (45%) children. From a societal perspective, the average cost per episode of RSV infection was USD 69 (95% confidence interval (CI) = 60–77) for outpatients and USD 488 (95% CI = 374–601) for inpatients. We estimated health system costs exceeding USD 30 and USD 200 on average for outpatient and inpatient care, respectively. Out-of-pocket costs borne by households were USD 32, (95% CI = 24–40) for outpatient care and USD 213 (95% CI = 158–269) for inpatient care. Household costs associated with RSV were equivalent to 88% (outpatients) and 592% (inpatients) of the Ghanaian monthly minimum wage in 2023.

**Conclusions:**

In this urban tertiary setting, we found a significant economic burden of medically attended RSV, carried by the Ghanaian health system and households. Our findings will be valuable to stakeholders in evaluating emerging interventions for RSV prevention in Ghana and comparable countries.

Respiratory syncytial virus (RSV) is a leading cause of acute lower respiratory tract infections (ALRIs), particularly in infants and young children, causing a considerable health burden. More than three million RSV-ALRI hospital admissions and over 100 000 RSV-attributable deaths are estimated to occur in children <5 years old each year globally. Children in low- and middle-income countries (LMICs) are disproportionately affected and account for 97% of RSV mortality [1]. This burden is especially pronounced in regions with high rates of poverty due to factors such as overcrowding, malnutrition, and limited access to healthcare [2,3]. In a study on medically attended ALRIs conducted in Ghana, a lower middle-income country at the time of writing, RSV was detected in 23% of children <5 years old and 33% of children <1 year old, in 2006 and 2013–2014 [4]. A more recent study on the burden of RSV at intensive care units reported 38% RSV positivity in children <2 years old [5].

Recent developments have led to the approval of two novel interventions for RSV prevention, with more products in the pipeline [6]. A long-acting monoclonal antibody [7] and a bivalent RSV pre-fusion protein maternal vaccine [8] have been recommended for global implementation by the Strategic Advisory Group of Experts on Immunisation (SAGE) of the World Health Organization (WHO) [9]. Gavi, The Vaccine Alliance prioritised RSV prevention in their 2018 Vaccine Investment Strategy [10]. Decisions to introduce RSV preventive strategies in LMICs, such as Ghana, will need to be guided by informed evaluations considering their potential impact on the health and economic burden of RSV.

RSV poses a substantial economic burden on health systems and households around the world. Early childhood RSV-ALRI is estimated to result in over USD 5 billion in direct medical costs, in 2017 [11]. However, limited country-specific data on the health and economic burden of RSV are available for LMICs, with a systematic review published in 2020 identifying none from the WHO African region [11]. A more recent systematic review included cost data from only one country in the region, Malawi, highlighting this persistent data limitation and showed a great variance in RSV-associated costs across countries [12]. At the time of writing, such data were available for Malawi, South Africa, and Kenya, among sub-Saharan African countries [13-15]. These studies reported a wide range of estimates for RSV costs of illness, which all posed a considerable economic burden within their contexts. To ascertain comparable data and inform stakeholders in evaluating RSV preventive interventions for Ghana, we estimated the health and economic burden of medically attended RSV infection in children <2 years old who sought care at a tertiary facility in Accra during one entire RSV season (2023).

## METHODS

### Study setting

We conducted this research as part of RSV GOLD III – Health Economics Study [16-18], a multi-country collaboration evaluating the economic burden of RSV and the impact and cost-effectiveness of RSV prevention in Gavi-eligible countries. This collaboration was an extension of the RSV GOLD III–ICU Network Study [5] and was based at several of the associated institutions, including the Department of Child Health at the Korle Bu Teaching Hospital (KBTH) located in the capital city, Accra, Ghana. The KBTH is a public tertiary referral centre serving most of the southern part of Ghana. It is the third largest referral centre in Africa with approximately 2000 beds and 21 clinical and diagnostic departments. The Department of Child Health at the KBTH includes an outpatient service, emergency department, and inpatient wards. All admissions to the wards are triaged via the emergency department, which sees about 25 000 patients with various health conditions per year.

Ghana’s Ministry of Health oversees public, private, and traditional healthcare organisations. Public facilities funded by the government play a significant role in providing healthcare services across primary (basic services and preventive care), secondary (specialised care and treatment), and tertiary (advanced specialised services and treatments) levels. In 2003 Ghana became the first African country to introduce a National Health Insurance Scheme (NHIS), with the aim of achieving universal health coverage [19]. By the end of 2021, 54% of Ghana’s population had active NHIS memberships [20]. In the same year, an estimated 27% of the Ghanaian healthcare expenditure was out-of-pocket [21]. Several private health insurance schemes complement the NHIS and provide additional coverage options for individuals and families.

### Study population

Children eligible for participation in this study were <2 years old and satisfied the WHO extended (severe) acute respiratory infection ((S)ARI) case definition [22]. (S)ARI was characterised by respiratory symptoms such as cough or shortness of breath, with an onset of symptoms within 10 days prior to the visit or admission. ARI case definition was adhered to for outpatient inclusions. The severity criterion applied to inpatient inclusions and was defined as ARI requiring hospitalisation. Children <4 days old and those who presented with non-respiratory symptoms or known non-infectious respiratory symptoms were excluded from the study.

Patient recruitment occurred at the outpatient department, emergency department, general ward, and paediatric ICU. At the KBTH, all inpatients were first observed at the emergency department. Due to the triage process at the study hospital, we defined outpatient care as any services received at the outpatient department or emergency department without overnight stay, and inpatient care as any services received at the emergency department over 24 hours or longer, or at any of the other inpatient wards.

### Data collection

Eligible children were prospectively recruited from June to November 2023, the period known to have a high incidence of RSV infection, characterising the RSV season in Ghana [4]. Caregivers provided informed consent and trained healthcare staff collected a nasopharyngeal sample on the day of presentation to the study facility, or the following Monday for weekend admissions. Samples were tested for RSV using molecular point-of-care testing (ID NOW RSV, Abbott, Scarborough, Maine, USA) [23]. This testing method had previously been validated at the same study facility [5]. Testing occurred within 72 hours of sampling and often on the same day.

Upon inclusion to the study, trained research staff obtained the patient’s demographic, socioeconomic, and clinical data from caregivers through questionnaire-led interviews. These questionnaires also ascertained out-of-pocket costs incurred for prior care for the same episode of illness as well as the index outpatient visit or admission. Hospital records provided clinical data. After discharge, detailed billing data were extracted from hospital records to capture costs incurred over the whole length of stay. During a follow-up interview, mostly conducted over telephone four to six weeks post discharge, caregivers were prompted to report costs incurred after the index visit or admission (Figure S1 in the **Online Supplementary Document**). For some children, caregivers sought certain services and medications outside of the study facility (*e.g.* at private facilities) during the length of stay. In these cases, caregivers presented receipts for data extraction. Overall, when feasible, caregiver reports were validated against hospital records and billing data, of which the latter two were prioritised in cases of discrepancies. All data were entered into the Castor electronic data capture system [24] where they were continuously monitored for quality and completeness assurance. No additional methods were needed to address missing data.

### Cost data

We described costs associated with one episode of (S)ARI from the societal, health system, and household perspectives, and grouped them into the following major categories:

1. Direct medical costs, such as costs for hospitality/facility-based fees, diagnostics including laboratory tests and imaging, as well as treatment and therapeutics including medication and respiratory support.
2. Direct non-medical costs, such as costs for transportation, meals, lodging, alternative caregivers, and childcare supplies.
3. Indirect costs, defined as lost income, productivity, and leisure time.

We excluded costs for diagnostics, treatment, and therapeutics not related to respiratory illness from the analysis.

Hospitality/facility-based fees included overhead costs (such as utility and wage expenses) sourced from hospital administration. Due to a multi-source wage structure at KBTH, we were unable to discern the total costs of wages for all staff at the Department of Child Health. Instead, we used the thirteenth month compensation budget as a proxy for average monthly wages and extrapolated them to an annual basis. Furthermore, overhead cost data were only available as aggregate departmental budgetary data for the Department of Child Health; therefore, we assumed equal costs per patient day for outpatients and inpatients. For inpatients, we multiplied the overhead cost per patient day by the individual length of stay to approximate overhead cost per admission. Other costs in this category (including consultation, bed, and maintenance fees) were paid out-of-pocket or by NHIS. To account for hospital revenue and prevent overlapping cost data, we deducted fees paid out-of-pocket or by NHIS from overhead costs for each patient individually.

We recorded the remaining direct medical costs as the product of unit cost and utilisation frequency for all services and products related to the episode of respiratory illness. We disaggregated these costs by the different sources of payment, including households (out-of-pocket components) and NHIS. Direct non-medical costs were caregiver-reported. For households reporting using their own car, we estimated transport costs by using travel distance and average fuel consumption for a return trip per facility visit. Caregivers reported lost income, while lost productivity and leisure time were monetised using the Ghanaian minimum wage in 2023 (GH₵ 402, equivalent to USD 36, per month) [25]. Costs were measured in GH₵ and converted to USD using the annual average exchange rate for 2023 (USD 1 = GH₵ 11.02) [26].

### Data analyses

This study described the demographic, socioeconomic, and clinical characteristics of RSV-positive and RSV-negative children by admission status (outpatient and inpatient cases). Using a bottom-up costing approach, we estimated costs associated with one episode of RSV- and non-RSV (S)ARI. We summarised costs as medians with interquartile ranges (IQRs) and means with 95% confidence intervals (CIs). We used bootstrapping (1000 replications) to estimate 95% CIs, which we zero-censored due to the non-negative nature of cost data. We estimated the total average direct medical, direct non-medical, and indirect costs by subcategories. To compare characteristics and costs across patient groups we used Wilcoxon rank-sum tests and applied a significance level of 5%. We conducted all statistical analyses separately for RSV-positive and RSV-negative patients using Stata, version 17.0 (Stata Corporation LLC, College Station, TX, USA) and visualised results using R, version 4.5.2 (R Foundation for Statistical Computing, Vienna, Austria) *via* RStudio, version 24.04. (Posit Software, PBC, Boston, Massachusetts, USA).

## RESULTS

### Participant characteristics

**Figure 1:**
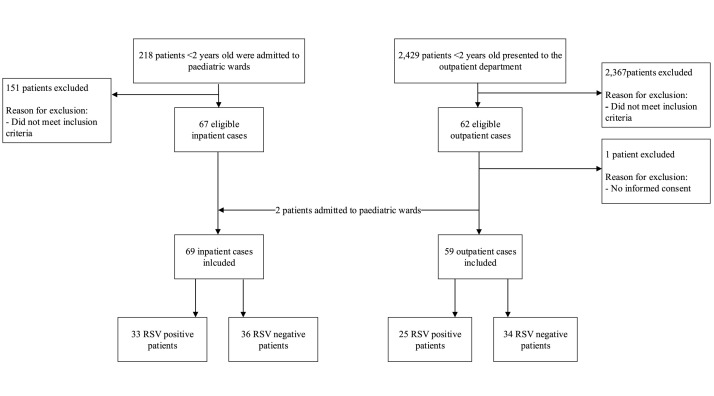
Flow diagram of patient inclusion. RSV – respiratory syncytial virus.

We identified 129 eligible patients during the respiratory season between June and November 2023 (Figure S2 in the **Online Supplementary Document**). As caregivers of all but one provided consent for participation (**Figure 1**), we included 128 children in our analysis, most of whom were accompanied by their mothers (n/N = 121/128, 95%), and approximately half of whom (n/N = 69/128, 54%) were admitted for inpatient care. RSV was detected in 45% (n/N = 58/128), with a slightly higher RSV-positivity observed in inpatients (n/N = 33/69, 48%) than outpatients (n/N = 25/59, 42%). Half of the participating children were <4 months old and most were term born and previously healthy (**Table 1**). The children who tested positive for RSV were significantly younger than their RSV-negative counterparts (median age two *vs*. four months, respectively) and age distributions differed significantly across these groups (*P* = 0.01). The most frequent diagnosis for outpatients was upper respiratory tract infection. Most inpatients were diagnosed with pneumonia and almost a third of them received respiratory support. One child was lost to follow-up (0.8%; RSV-negative; inpatient) and we observed two in-hospital fatalities (one RSV-positive).

**Table 1:** Demographic, socioeconomic, and clinical characteristics of participating children younger than two years <2 years old by RSV and admission status*

	RSV-positive			RSV-negative			All patients
	**Total**	**Outpatient**	**Inpatient**	**Total**	**Outpatient**	**Inpatient**	**Total**
**Total number of participants**	58	25	33	70	34	36	128
**Age in months, MD (IQR)**	2 (1–5)	3 (1–4)	2 (1–5)	4 (1–12)	6 (1–13)	4 (1–11)	3 (1–8)
**Female**	28 (48.3)	10 (40.0)	18 (54.6)	25 (35.7)	15 (44.1)	10 (27.8)	53 (41.4)
**Household size, MD (IQR)**	4 (3–6)	4 (3–5)	4 (4–6)	5 (3–5)	4 (3–5)	5 (4–6)	5 (3–5)
**Level of education primary caregiver secondary school or higher**	36 (62.1)	15 (60.0)	21 (63.6)	41 (58.6)	21 (61.8)	20 (55.6)	77 (60.2)
**Health insurance**	48 (82.8)	18 (72.0)	30 (90.9)	57 (81.4)	26 (76.5)	31 (86.1)	105 (82.0)
**Distance to healthcare facility in km, MD (IQR)**	8.2 (4.1–14.2)	8.5 (5.6–13.0)	7.4 (4.0–15.0)	9.1 (4.1–21.0)	6.7 (4.0–18.6)	10.4 (4.6–22.5)	8.4 (4.1–19.0)
**Diagnosis at admission**							
Bronchiolitis	5 (8.6)	1 (4.0)	4 (12.1)	6 (8.6)	2 (5.9)	4 (11.1)	11 (8.6)
Pneumonia	28 (48.3)	2 (8.0)	26 (78.8)	26 (37.1)	3 (8.8)	23 (63.9)	54 (42.2)
URTI	24 (41.4)	22 (88.0)	2 (6.1)	29 (41.4)	25 (73.5)	4 (11.1)	53 (41.4)
Other	1 (1.7)	-	1 (3.0)	9 (12.9)	4 (11.8)	5 (13.9)	10 (7.8)
**Respiratory support**	21 (36.2)	-	21 (63.6)	17 (24.3)	-	17 (47.2)	38 (29.7)
**Prematurity**	14 (24.1)	4 (16.0)	10 (30.3)	17 (24.3)	11 (32.4)	6 (16.7)	31 (24.2)
**Comorbidity**	12 (20.7)	4 (16.0)	8 (24.2)	23 (32.9)	7 (20.6)	16 (44.4)	35 (27.3)
**Length of stay in days, MD (IQR)**	6.0 (4.0–7.0)	-	6.0 (4.0–7.0)	5.0 (2.0–9.0)	-	5.0 (2.0–9.0)	6.0 (3.0–8.0)
**Prior medical consultation**	27 (46.6)	8 (32.0)	19 (57.6)	30 (42.9)	14 (41.2)	16 (44.4)	57 (44.5)
**Follow-up care**	8 (13.8)	2 (8.0)	6 (18.2)	10 (14.5)†	5 (14.7)	5 (14.3)†	18 (14.1)†
**Mortality**	1 (1.7)	-	1 (3.0)	1 (1.4)†	-	1 (2.8)†	2 (1.6)†

IQR – interquartile range, MD – median, RSV – respiratory syncytial virus, URTI – upper respiratory tract infection

*Values presented as n (%) unless specified otherwise.

†One patient was lost to follow up, leaving 69 RSV-negative patients, 35 RSV-negative inpatients, and 127 patients overall.

### Societal costs

**Figure 2:**
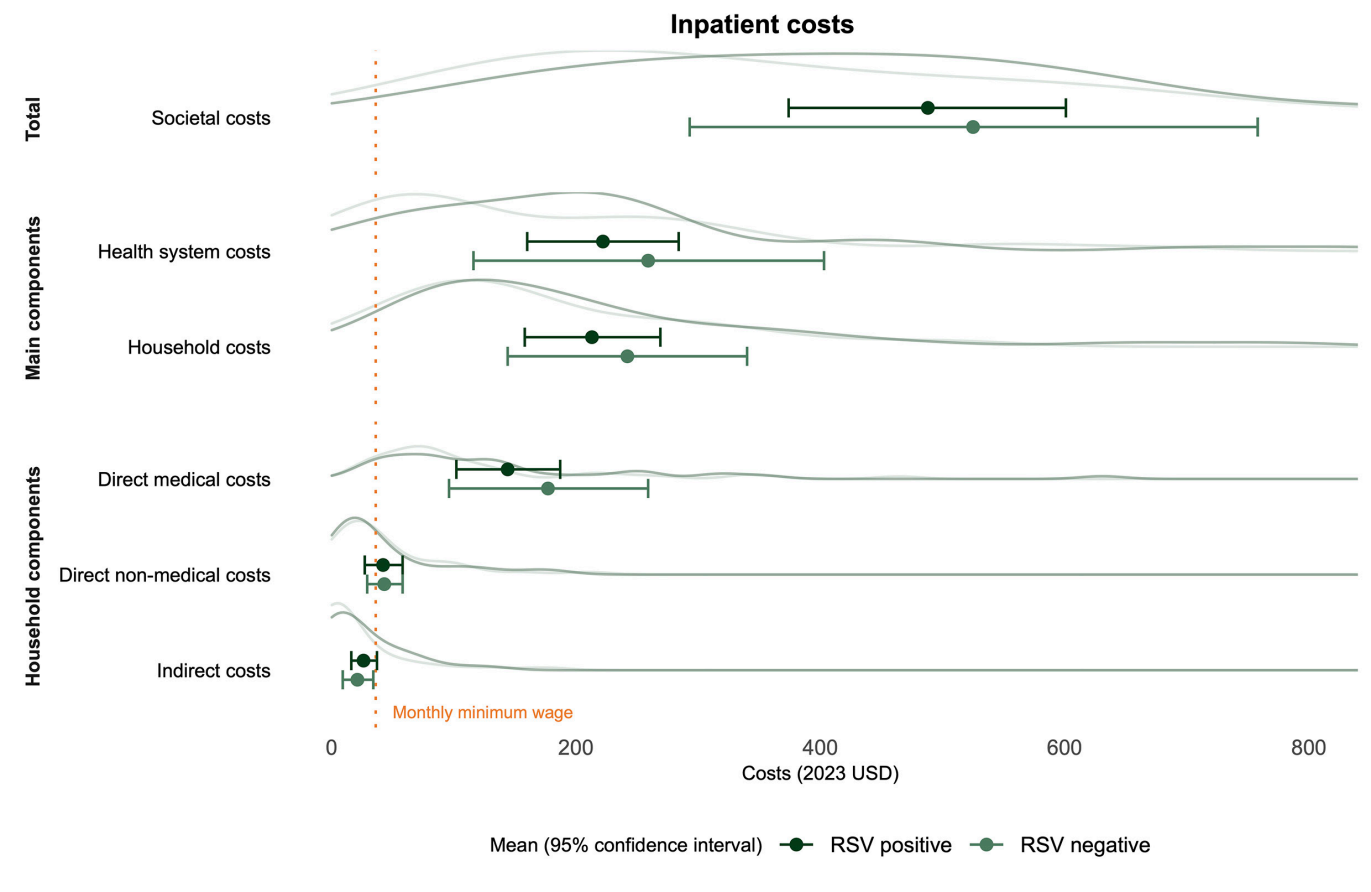
Mean costs of RSV and non-RSV severe acute respiratory illness among children <2 years old seeking care at Korle Bu Teaching Hospital in Accra, Ghana across various cost categories. The density curves illustrate the distribution of the underlying data. Costs varied widely across patients, and distributions extended beyond USD 800 (9/69 patients had total costs: USD 829; 887, 911; 1035; 1111; 1201; 1486; 1585; and 4427). For reference, the monthly minimum wage at the time of study is indicated by the orange vertical dotted line. RSV – respiratory syncytial virus.

Total costs for both RSV and non-RSV (S)ARI varied widely (**Figure 2**). Total societal costs consisted mainly of direct medical costs and were similar between RSV-positive and RSV-negative cases. On average, one episode of RSV infection resulted in USD 69 (95% CI = 60–77) for children receiving outpatient care and USD 488 (95% CI = 374–601) for children receiving inpatient care (**Table 2**). These costs included costs for the index visit or admission, as well as prior and follow-up care. When excluding prior and follow-up care, the average costs per outpatient visit and hospital admission were USD 61 (95% CI = 55–67) and USD 462 (95% CI = 354–570), respectively, for children with RSV (Table S1 in the **Online Supplementary Document**).

**Table 2:** Summary of societal costs per episode of (severe) acute respiratory infection in children <2 years old by RSV and admission status, expressed in 2023 USD

	RSV-positive (n = 58)		RSV-negative (n = 70)	
	**Outpatient (n = 25)**	**Inpatient (n = 33)**	**Outpatient (n = 34)**	**Inpatient (n = 36)**
**Societal costs total**				
x̅ (95% CI)	68.58 (60.19–76.96)	487.66 (374.04–601.29)	106.33 (74.12–138.54)	525.37 (292.67–758.07)
MD (IQR)	68.57 (51.84–78.36)	422.84 (272.52–598.28)	66.46 (52.15–114.79)	345.74 (184.27–593.66)
Direct medical costs				
x̅ (95% CI)	55.26 (49.69–60.82)	366.53 (269.81–463.25)	70.91 (51.89–89.93)	436.81 (232.35–641.27)
MD (IQR)	52.26 (43.27–67.25)	278.56 (189.72–428.52)	53.92 (38.34–72.62)	269.62 (136.64–491.88)
*Hospitality/facility-based fees*				
x̅ (95% CI)	37.59 (36.15–39.03)	239.14 (177.87–300.41)	37.59 (36.76–38.42)	291.40 (148.48–434.32)
MD (IQR)	36.02 (36.02–36.02)	216.09 (144.06–254.83)	36.02 (36.02–38.74)	180.08 (83.73–324.14)
*Medication costs*				
x̅ (95% CI)	8.41 (5.54–11.28)	45.66 (36.12–55.19)	16.50 (8.27–24.73)	56.24 (25.24–87.23)
MD (IQR)	6.26 (2.54–12.07)	38.79 (24.04–73.37)	9.80 (1.91–20.31)	27.54 (21.36–53.74)
*Laboratory costs*				
x̅ (95% CI)	12.54 (8.53–16.55)	23.49 (18.69–28.30)	18.14 (9.31–26.96)	24.85 (19.71–29.99)
MD (IQR)	13.70 (7.26–16.42)	18.75 (13.70–31.90)	14.93 (7.26–21.78)	20.10 (14.34–33.12)
*Imaging costs*				
x̅ (95% CI)	0.85 (0.00–2.48)	6.51 (3.90–9.12)	5.53 (0.00–11.33)	11.43 (3.14–19.71)
MD (IQR)	0.00 (0.00–0.00)	5.04 (5.04–6.81)	0.00 (0.00–6.81)	6.17 (5.04–10.08)
*Procedure costs*				
x̅ (95% CI)	-	61.49 (22.94–100.05)	8.51 (0.00–19.84)	88.90 (18.53–159.28)
MD (IQR)	-	21.78 (0.00–89.84)	0.00 (0.00–0.00)	21.78 (0.00–107.08)
*Miscellaneous costs*				
x̅ (95% CI)	0.35 (0.00–1.06)	6.45 (5.13–7.76)	1.11 (0.00–2.34)	6.61 (4.26–8.97)
MD (IQR)	0.00 (0.00–0.00)	5.44 (2.72–9.30)	0.00 (0.00–0.00)	5.44 (2.72–7.26)
Direct non-medical costs*				
x̅ (95% CI)	7.74 (4.31–11.17)	42.31 (26.53–58.08)	9.88 (5.66–14.10)	43.47 (28.73–58.22)
MD (IQR)	5.44 (3.63–8.24)	21.78 (15.43–54.99)	4.45 (2.72–10.89)	28.58 (15.43–52.00)
*Transport costs*				
x̅ (95% CI)	6.90 (3.91–9.89)	12.50 (4.69–20.31)	6.28 (3.99–8.57)	8.59 (5.23–11.95)
MD (IQR)	5.44 (2.72–7.26)	4.54 (1.63–11.59)	3.63 (2.54–7.62)	4.54 (2.01–9.62)
*Meal costs*				
x̅ (95% CI)	0.85 (0.22–1.47)	29.81 (18.59–41.03)	3.33 (0.60–6.05)	33.89 (19.87–47.92)
MD (IQR)	0.00 (0.00–0.91)	18.15 (8.17–38.11)	0.00 (0.00–1.81)	18.15 (10.89–38.11)
Indirect costs†				
x̅ (95% CI)	5.58 (0.67–10.49)	78.83 (46.94–110.72)	25.55 (8.91–42.18)	45.09 (16.77–73.41)
MD (IQR)	0.00 (0.00–8.44)	34.81 (6.75–112.27)	1.28 (0.00–23.74)	4.73 (0.00–39.02)
*Lost productivity*				
x̅ (95% CI)	4.03 (0.24–7.82)	52.37 (28.02–76.72)	12.21 (4.46–19.96)	23.68 (7.48–39.87)
MD (IQR)	0.00 (0.00–3.97)	28.92 (0.00–61.31)	0.79 (0.00–14.51)	0.00 (0.00–29.61)
*Lost income*				
x̅ (95% CI)	1.52 (0.17–2.88)	19.84 (10.26–29.43)	13.16 (3.23–23.08)	14.62 (4.93–24.31)
MD (IQR)	0.00 (0.00–0.00)	0.00 (0.00–36.30)	0.00 (0.00–7.26)	0.00 (0.00–9.07)
*Lost leisure*				
x̅ (95% CI)	0.03 (0.00–0.08)	6.62 (3.99–9.25)	0.18 (0.02–0.34)	6.79 (1.33–12.24)
MD (IQR)	0.00 (0.00–0.00)	4.05 (0.00–9.45)	0.00 (0.00–0.00)	1.35 (0.00–7.43)

CI – confidence interval, IQR – interquartile range, MD – median, RSV – respiratory syncytial virus, x̄ – mean

*Additional to transport and meal costs, we measured lodging costs and caregiver costs. Only one caregiver reported lodging costs (USD 16) and none of the caregivers reported costs for alternative caregivers in their absence while caring for the ill child.

†Lost income was self-reported by caregivers, while both productivity loss and lost leisure were monetised using the Ghanaian minimum wage (USD 36 per month) and the time absent from work and lost leisure time while caring for the ill child, respectively.

### Health system costs

From the health system perspective, average costs included direct medical costs only and were mainly driven by hospitality/facility-based fees. The average total health system cost per episode of RSV infection was USD 33 (95% CI = 32–34) for outpatients and USD 222 (95% CI = 160–284) for inpatients. Despite high reporting of NHIS membership (n/N = 105/128, 82%), costs covered by NHIS were negligible. NHIS contributions were mostly for hospitality/facility-based fees (n/N = 85/128, 66%) and additionally observed for laboratory (n/N = 11/128, 9%) and imaging (n/N = 26/128, 20%) services (**Table 3**). No costs were covered by alternative (private) insurances in this study.

**Table 3:** Summary of health system and household costs per episode of (severe) acute respiratory infection in children <2 years old by RSV and admission status, expressed in 2023 USD

	RSV-positive (n = 58)		RSV-negative (n = 70)	
	**Outpatient (n = 25)**	**Inpatient (n = 33)**	**Outpatient (n = 34)**	**Outpatient (n = 36)**
Health system costs total*				
x̅ (95% CI)	32.86 (32.01–33.71)	222.03 (160.09–283.98)	31.95 (31.04–32.86)	259.33 (116.05–402.61)
MD (IQR)	33.29 (33.29–33.29)	205.98 (101.21–251.52)	33.29 (30.63–33.29)	138.79 (65.50–283.06)
Hospitality/facility-based fees				
x̅ (95% CI)	32.86 (32.01–33.71)	219.22 (158.22–280.22)	31.93 (31.02–32.84)	257.34 (114.54–400.15)
MD (IQR)	33.29 (33.29–33.29)	205.98 (100.61–246.48)	33.29 (30.63–33.29)	139.29 (64.59–281.05)
Laboratory costs				
x̅ (95% CI)	-	0.13 (0.00–0.25)	0.03 (0.00–0.10)	0.21 (0.00–0.43)
MD (IQR)	-	0.00 (0.00–0.00)	0.00 (0.00–0.00)	0.00 (0.00–0.00)
Imaging costs				
x̅ (95% CI)	-	2.87 (1.60–4.14)	-	2.67 (1.28–4.06)
MD (IQR)	-	0.00 (0.00–5.04)	-	0.00 (0.00–5.04)
**Household costs total**				
x̅ (95% CI)	31.69 (23.70–39.68)	213.26 (157.75–268.78)	62.17 (34.17–90.17)	242.36 (144.49–340.23)
MD (IQR)	29.44 (15.43–37.43)	172.75 (99.88–264.20)	28.70 (12.52–71.05)	139.97 (95.56–276.73)
Direct medical costs				
x̅ (95% CI)	22.39 (16.76–28.03)	144.49 (102.41–186.58)	38.96 (19.84–58.07)	177.48 (95.78–259.18)
MD (IQR)	20.69 (9.98–32.21)	112.01 (63.93–177.00)	21.96 (5.95–39.33)	84.92 (64.49–173.09)
*Hospitality/facility-based fees*				
x̅ (95% CI)	4.73 (3.28–6.18)	19.92 (7.58–32.26)	5.66 (4.23–7.09)	34.06 (9.40–58.71)
MD (IQR)	2.72 (2.72–5.39)	8.80 (7.44–14.65)	5.39 (2.72–5.44)	10.39 (7.03–34.30)
*Medication costs*				
x̅ (95% CI)	8.41 (5.54–11.28)	45.66 (36.12–55.19)	16.50 (8.27–24.73)	56.24 (25.24–87.23)
MD (IQR)	6.26 (2.54–12.07)	38.79 (24.04–73.37)	9.80 (1.91–20.31)	27.54 (21.36–53.74)
*Laboratory costs*				
x̅ (95% CI)	12.54 (8.53–16.55)	24.12 (19.30–28.94)	18.10 (9.29–26.92)	24.64 (19.45–29.82)
MD (IQR)	13.70 (7.26–16.42)	19.33 (13.70–33.67)	14.93 (7.26–21.78)	20.10 (13.70–33.12)
*Imaging costs*				
x̅ (95% CI)	0.85 (0.00–2.48)	3.64 (1.39–5.90)	5.53 (0.00–11.33)	8.76 (0.85–16.67)
MD (IQR)	0.00 (0.00–0.00)	0.00 (0.00–6.81)	0.00 (0.00–6.81)	0.00 (0.00–6.81)
*Procedure costs*				
x̅ (95% CI)	-	61.49 (22.94–100.05)	8.51 (0.00–19.84)	88.90 (18.53–159.28)
MD (IQR)	-	21.78 (0.00–89.84)	0.00 (0.00–0.00)	21.78 (0.00–107.08)
*Miscellaneous costs*				
x̅ (95% CI)	0.35 (0.00–1.06)	6.45 (5.13–7.76)	1.11 (0.00–2.34)	6.61 (4.26–8.97)
MD (IQR)	0.00 (0.00–0.00)	5.44 (2.72–9.30)	0.00 (0.00–0.00)	5.44 (2.72–7.26)
Direct non-medical costs†				
x̅ (95% CI)	7.74 (4.31–11.17)	42.31 (26.53–58.08)	9.88 (5.66–14.10)	43.47 (28.73–58.22)
MD (IQR)	5.44 (3.63–8.24)	21.78 (15.43–54.99)	4.45 (2.72–10.89)	28.58 (15.43–52.00)
*Transport costs*				
x̅ (95% CI)	6.90 (3.91–9.89)	12.50 (4.69–20.31)	6.28 (3.99–8.57)	8.59 (5.23–11.95)
MD (IQR)	5.44 (2.72–7.26)	4.54 (1.63–11.59)	3.63 (2.54–7.62)	4.54 (2.01–9.62)
*Meal costs*				
x̅ (95% CI)	0.85 (0.22–1.47)	29.81 (18.59–41.03)	3.33 (0.60–6.05)	33.89 (19.87–47.92)
MD (IQR)	0.00 (0.00–0.91)	18.15 (8.17–38.11)	0.00 (0.00–1.81)	18.15 (10.89–38.11)
Indirect costs‡				
x̅ (95% CI)	1.55 (0.20–2.91)	26.46 (15.55–37.37)	13.34 (3.33–23.35)	21.41 (8.53–34.29)
MD (IQR)	0.00 (0.00–0.00)	12.15 (1.35–40.35)	0.00 (0.00–7.26)	4.05 (0.00–19.58)
*Lost income*				
x̅ (95% CI)	1.52 (0.17–2.88)	19.84 (10.26–29.43)	13.16 (3.23–23.08)	14.62 (4.93–24.31)
MD (IQR)	0.00 (0.00–0.00)	0.00 (0.00–36.30)	0.00 (0.00–7.26)	0.00 (0.00–9.07)
*Lost leisure*				
x̅ (95% CI)	0.03 (0.00–0.08)	6.62 (3.99–9.25)	0.18 (0.02–0.34)	6.79 (1.33–12.24)
MD (IQR)	0.00 (0.00–0.00)	4.05 (0.00–9.45)	0.00 (0.00–0.00)	1.35 (0.00–7.43)

CI – confidence interval, IQR – interquartile range, MD – median, RSV – respiratory syncytial virus, x̄ – mean

*Health system costs included direct medical costs only. Additional to the listed costs, direct medical costs included costs for medications, procedures, and miscellaneous costs. We did not observe any of these costs from the health system perspective.

†Additional to transport and meal costs, we measured lodging costs and caregiver costs. Only one caregiver reported lodging costs (USD 16) and none of the caregivers reported costs for alternative caregivers in their absence while caring for the ill child.

‡Lost income was self-reported by caregivers, while lost leisure was monetised using the Ghanaian minimum wage (USD 36 per month) and lost leisure time while caring for the ill child.

### Household costs

Per episode of RSV infection, households incurred average costs of USD 32 (95% CI = 24–40) and USD 213 (95% CI = 158–269) for outpatient and inpatient cases, respectively (**Table 3**). Direct medical costs for outpatients consisted mainly of laboratory costs, while for inpatients the main components were costs for procedures including oxygen therapy and medications. Indirect costs applied for 52% (n/N = 66/128) of households, including 42 (64%) caregivers who reported lost income as a result of caring for an ill child. All caregivers accompanying the child were asked to report their income. Overall, interviewed caregiver(s) of 96 of the 128 (75%) children had a monthly income. Of these, household costs for all-cause ARI were more than 20% of the caregiver monthly income for 71% (n/N = 68/96) and exceeded caregiver monthly income for 28% (n/N = 27/96, data not shown). Most caregivers reported using personal savings (100% of outpatients; n/N = 65/69 (94%) of inpatients,) to cover out-of-pocket costs.

## DISCUSSION

The global cost of RSV-related illness was estimated at over USD 5 billion in 2017, 65% of which was incurred in LMICs [11]. Country-specific data on the economic burden of RSV are limited for LMICs [12]. To our knowledge, this was the first prospective study to estimate specific costs associated with RSV infection in Ghana. We observed an entire local RSV season and described the socioeconomic and clinical characteristics of children <2 years old, who sought outpatient and inpatient care for (S)ARIs at an urban tertiary facility. We estimated average direct medical, direct non-medical, and indirect costs from the societal, health system, and household perspectives.

We observed higher RSV positivity than noted in previous research at the same facility [4,5]. This discrepancy may be due to differences in inclusion criteria or temporal differences in seasonal peaks. We found that RSV was more prevalent among admitted children (48%) than children visiting the outpatient ward (43%) and described patients’ characteristics according to RSV and admission status. This study achieved a high recruitment rate, with only one patient lost to follow-up and was, therefore, less likely affected by selection biases.

In our analysis, RSV was associated with societal costs equivalent to 3% (USD 69; 4%, USD 106, for non-RSV (S)ARI) and 20% (USD 488; 22%, USD 525, for non-RSV (S)ARI) of the 2023 Ghanaian gross domestic product (GDP) per capita, for outpatient and inpatient care, respectively [27]. Our findings were comparable to those of a previous study conducted in children <5 years old in Kenya, where RSV was estimated to cost USD 300 (15% of GDP per capita) and USD 479 (25% of GDP per capita) for inpatient care at two semi-rural referral hospitals in 2019–2021 (adjusted for inflation in local currency and converted to 2023 USD) [15,28]. The Kenyan study was the only known comparable study conducted in a lower-middle income sub-Saharan African country.

Health system costs estimated here depended on the assumption that the thirteenth month compensation budgeted for the participating facility reflected the average costs of wages for all staff. While this assumption is a potential limitation of this study due to substantial uncertainty, it was the nearest available alternative to facilitate an estimate of overall healthcare delivery costs due to shortages in data for human resources costs. We have reported cost estimates across categories, many of which are unaffected by this assumption, thereby providing a comprehensive set of estimates that may be reliably extracted for future studies. Similar studies conducted in the future may address this gap by prospectively collecting wage costs data.

Although most (n/N = 105/128, 82%) caregivers reported NHIS coverage, costs covered by NHIS were frequently negligible. The level of NHIS coverage observed in our sample far exceeded the national average, likely due to the urban study setting [19,20]. The observed low proportion of costs charged to NHIS may be indicative of potential reporting biases in the current study or inefficiencies in NHIS services in Ghana. The 2022 Ghana Demographic and Health Survey reported that men and women utilising healthcare services with insurance frequently faced out-of-pocket payments, both as co-pay and in full [29]. Our findings may reiterate this public health challenge, signalling a need for policy action to realise the aim of achieving universal health coverage in Ghana through the NHIS by alleviating out-of-pocket healthcare expenditure.

Most caregivers resorted to personal savings to cover costs of care for all-cause (S)ARI. When compared to reported income, household costs exceeded caregiver monthly income for 28% of the children whose caregivers reported an income (n/N = 27/96). Notably, only the income of caregivers accompanying the ill child was recorded. Since most children were accompanied by one caregiver (mostly mothers), this observation is unlikely to reflect the overall household income. Therefore, we were unable to estimate the proportion of households facing catastrophic healthcare expenditure. When compared to the national minimum wage (USD 36 per month), average household costs for outpatients and inpatients respectively corresponded to 88% and 592% for RSV-positive patients and 173% and 673% for RSV-negative patients. A limitation of our analysis is the use of self-reported data collected over several weeks. Most costs, however, were incurred during the index visit/admission and gathered from hospital records, therefore, mitigating the impact of potential recall bias. Similarly, where possible, patient characteristics were extracted from or verified in clinical records.

Given that costs of care depend on the available level of care and its affordability for the intended population, our findings are contingent on the urban and tertiary care setting of the study. A previous study based on the 2017 Ghana Living Standard Survey showed a strong influence of socioeconomic factors, including income on healthcare resource utilisation behaviour in the country [30]. By nature of the study facility, our analyses may have limited generalisability at the national level. Nonetheless, this study was the first of its kind in Ghana and offers critical insights into RSV-related costs that may inform policy and serve as inputs for modelling costs across other settings (such as primary healthcare centres in rural settings) to uncover nuances in RSV-related healthcare utilisation and the associated costs.

Pharmaceutical interventions to protect all infants against RSV-related illness in their first RSV season have emerged and many more remain in development [6]. Recently, WHO’s SAGE issued recommendations to implement these interventions globally [9]. Gavi has prioritised support for the introduction of these products in eligible countries including Ghana [10,31]. To realise these recommendations, international and national stakeholders will need to assess the value of introducing RSV preventive interventions on a country-level, considering their impact on the short- and long-term health burden, as well as the economic burden of early-life RSV disease. This study exposes a large health and economic burden of RSV, especially in the first months of life, which may be averted through immunisation. The findings presented above will inform evaluations of the potential impact of implementing RSV immunisation strategies to protect infants in Ghana and help estimate their cost-effectiveness.

## CONCLUSIONS

This study estimated substantial costs associated with episodes of RSV infection in children <2 years old seeking care for (S)ARI at a tertiary healthcare facility in Accra, Ghana. Findings presented above contribute to the growing body of data of the health and economic burden of RSV in LMICs. Given recent SAGE recommendations, (inter)national stakeholders may apply our findings to evaluate novel pharmaceutical strategies to protect infants in their first RSV season in Ghana and comparable settings.

## Additional material


Online Supplementary Document
