# Supplementary material for: The economic burden of respiratory syncytial virus and other acute respiratory infections among children <2 years old seeking care at a tertiary facility in Accra, Ghana
Source: J Glob Health. 2026 May 29;16:04040. doi: 10.7189/jogh.16.04040 (PMC13219970; doi:10.7189/jogh.16.04040)
Supplement: Online Supplementary Document [file jogh-16-04040-s001.pdf]

Supplement to: Shaaban FL, Dame J, Nguyen A, Rave N, Pecenka C, Bont L; RSV GOLD III – Health Economics Study Group. The economic burden of respiratory syncytial virus and other acute respiratory infections among children <2 years old seeking care at a tertiary facility in Accra, Ghana. J Glob Health. 2026;16:04040.

With the RSV GOLD III – Health Economics Study we evaluate costs associated with acute lower respiratory tract infections (LRTIs) in children <2 years old in four low- and middle-income countries (Ghana, Mozambique, Nepal, and Nigeria), during one local respiratory season. Here, we provide supplementary material related to the study conducted in Ghana.

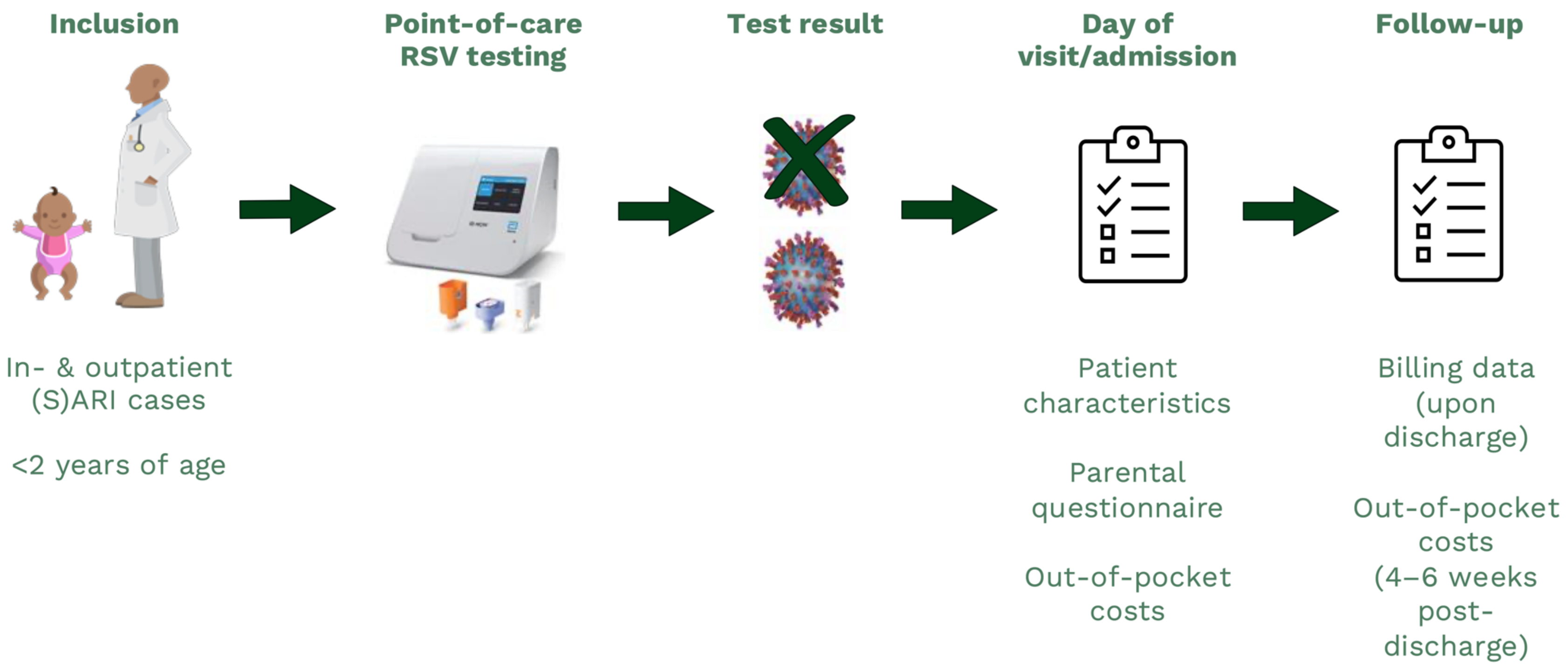

Figure S1. Study setup and data collection process.
RSV – respiratory syncytial virus, (S)ARI – (severe) acute respiratory infection.

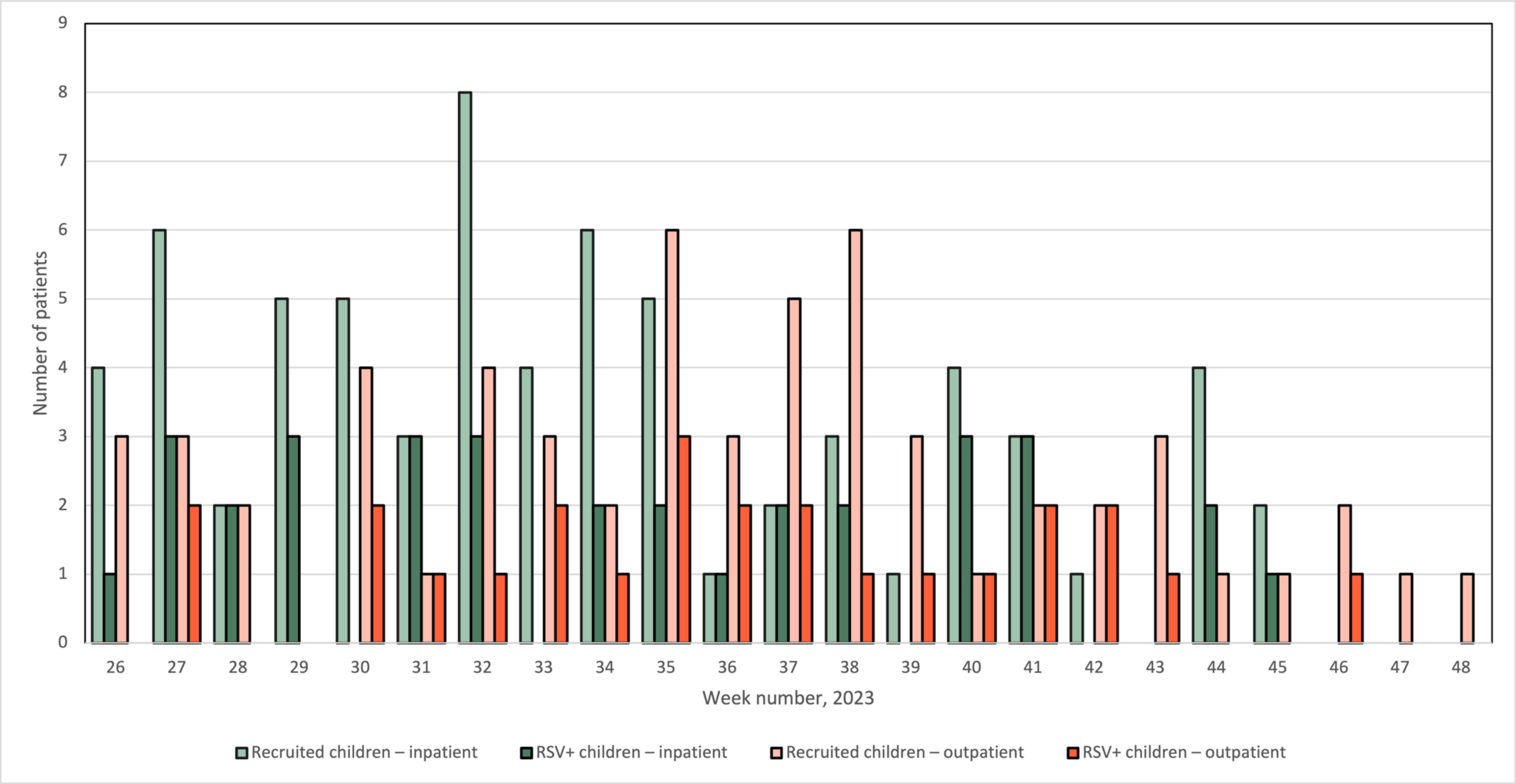

Figure S2. Weekly recruitment numbers during the study period at the Department of Child Health at Korle Bu Teaching Hospital in Accra, Ghana over one RSV season (June–November 2023) by admission status.
RSV – respiratory syncytial virus.

Table S1. Average total societal costs per episode of (severe) acute respiratory infection in children <2 years old by point of healthcare contact expressed in 2023 USD.

	RSV-positive (n=58)		RSV-negative (n=70)	
	**Outpatient (n=25)**	**Inpatient (n=33)**	**Outpatient (n=34)**	**Inpatient (n=36)**
**Prior medical consultation costs***				
$\bar{x}$ (95% CI)	4.33 (0.34–8.32)	22.03 (6.70–37.36)	20.49 (5.46–35.52)	29.28 (9.25–49.30)
MD (IQR)	0.00 (0.00–3.18)	1.81 (0.00–20.87)	0.00 (0.00–14.07)	0.00 (0.00–29.90)
**Index visit/admission costs**				
$\bar{x}$ (95% CI)	60.95 (54.55–67.36)	462.05 (354.16–569.94)	64.41 (56.59–72.23)	494.05 (261.21–726.90)
MD (IQR)	56.07 (48.72–71.69)	422.84 (256.46–522.87)	56.10 (44.62–77.54)	313.26 (156.01–534.68)
**Follow-up care costs†**				
$\bar{x}$ (95% CI)	3.30 (0.00–7.75)	3.59 (0.52–6.65)	21.43 (0.00–44.50)	2.10 (0.25–3.95)
MD (IQR)	0.00 (0.00–0.00)	0.00 (0.00–0.00)	0.00 (0.00–0.00)	0.00 (0.00–0.00)

*n=57/128 had a prior medical consultation.

†n=18/127 received follow-up care; n=1/128 was lost to follow-up and follow-up care status was unknown.

CI – confidence interval, IQR – interquartile range, MD – median, RSV – respiratory syncytial virus.

RSV GOLD III – Health Economics Study Group members*

Cameroon:

Frédéric Debellut – Center for Vaccine Innovation and Access, PATH, Geneva, Switzerland
Norbert Fuhngwa – Triangle Research Foundation, Douala, Cameroon
Henshaw Mandi – Triangle Research Foundation, Douala, Cameroon

Ghana:

Rosemary Akuaku – Department of Child Health, Korle Bu Teaching Hospital, Accra, Ghana
Joycelyn Dame – Department of Child Health, University of Ghana Medical School Korle Bu Teaching Hospital, Accra, Ghana
Amma Ekem – Department of Child Health, Korle Bu Teaching Hospital, Accra, Ghana
Bamenla Goka – Department of Child Health, University of Ghana Medical School Korle Bu Teaching Hospital, Accra, Ghana
Ebenezer Ntow – Department of Child Health, Korle Bu Teaching Hospital, Accra, Ghana
Kwabena A. Osman – Department of Child Health, University of Ghana Medical School Korle Bu Teaching Hospital, Accra, Ghana

Mozambique:

Assucênio Chissaque – Instituto Nacional de Saúde, Marracuene district, Maputo, Mozambique; Instituto de Higiene e Medicina Tropical, Universidade Nova de Lisboa, Lisbon, Portugal
Nilsa de Deus – Instituto Nacional de Saúde, Marracuene district, Maputo, Mozambique
Esperança Lourenço Guimarães – Instituto Nacional de Saúde, Marracuene district, Maputo, Mozambique; Instituto de Higiene e Medicina Tropical, Universidade Nova de Lisboa, Lisbon, Portugal
Braiton Maculuve – Ministério da Saúde, Maputo, Mozambique
Elias Manjate – Faculty of Medicine, University Eduardo Mondlane, Maputo, Mozambique
Yara Manjate – Faculty of Medicine, University Eduardo Mondlane, Maputo, Mozambique
Izilda Matimbe – Faculty of Medicine, University Eduardo Mondlane, Maputo, Mozambique
Tufária Mussá – Faculty of Medicine, University Eduardo Mondlane, Maputo, Mozambique
Mirela Pale – Instituto Nacional de Saúde, Marracuene district, Maputo, Mozambique
Cesar Palha – Faculty of Medicine, University Eduardo Mondlane, Maputo, Mozambique

Cristina Sinussene – Faculty of Medicine, University Eduardo Mondlane, Maputo, Mozambique
Farida Zavala – Faculty of Medicine, University Eduardo Mondlane, Maputo, Mozambique

Nepal:

Ram H. Chapagain – Kanti Children's Hospital, Kathmandu, Nepal; Nepal Paediatrics Society, Kathmandu, Nepal
Rita Dhital – Nepal Paediatrics Society, Kathmandu, Nepal
Upendra Dhungana – Public Health Administrator; Ministry of Health and Population. Nepal
Prakash Joshi – Kanti Children's Hospital, Kathmandu, Nepal; Nepal Paediatrics Society, Kathmandu, Nepal
Ranju Karki – Nepal Paediatrics Society, Kathmandu, Nepal
Adita Nepali – Nepal Paediatrics Society, Kathmandu, Nepal
Uttam Paudel – Post Doctorate Researcher (Health Economics), Chulalongkorn University
Arun K. Sharma – Tribhuvan University Teaching Hospital, Institute of Medicine, Kathmandu, Nepal; Nepal Paediatrics Society, Kathmandu, Nepal
Rupesh Shrestha – Tribhuvan University Teaching Hospital, Institute of Medicine, Kathmandu, Nepal
Nirasta Thakili – Nepal Paediatrics Society, Kathmandu, Nepal

Nigeria:

Fadlulai Abdu-Raheem – Department of Paediatrics, Ahmadu Bello University Teaching Hospital, Zaria, Nigeria
Anas Abubakar – Department of Paediatrics, Ahmadu Bello University Teaching Hospital, Zaria, Nigeria
Abdullahi Aminu – Department of Paediatrics, Ahmadu Bello University Teaching Hospital, Zaria, Nigeria
Maria A. Garba – Department of Paediatrics, Ahmadu Bello University Teaching Hospital, Zaria, Nigeria
Fatima J. Giwa – Department of Medical Microbiology, Ahmadu Bello University Teaching Hospital, Zaria, Nigeria
Habiba Lawal – Institute of Child Health, Ahmadu Bello University, Banzazzau, Zaria, Nigeria
Bernsah D. Lawong – Department of Economics, Ahmadu Bello University, Zaria, Nigeria
Abdullahi Musa – Department of Paediatrics, Ahmadu Bello University Teaching Hospital, Zaria, Nigeria
Teddy Naddumba – Center for Vaccine Innovation and Access, PATH, Kampala, Uganda
Aira A. Olorukooba – Department of Paediatrics, Ahmadu Bello University Teaching Hospital, Zaria, Nigeria

Support:
Andrew Clark – Department of Health Services Research and Policy, London School of Hygiene & Tropical Medicine, London, UK
An Nguyen – Center for Vaccine Innovation and Access, PATH, Ho Chi Minh city, Vietnam
Clint Pecenka – Center for Vaccine Innovation and Access, PATH, Seattle, WA, USA

The Netherlands:
Louis J. Bont – University Medical Centre Utrecht, Utrecht, The Netherlands
Neele Rave – University Medical Centre Utrecht, Utrecht, The Netherlands
Farina L. Shaaban – University Medical Centre Utrecht, Utrecht, The Netherlands

*The authors are listed in alphabetical order of their surnames, according to the specific country teams through which they were involved in the study. Team members from University Medical Centre Utrecht, along with supporting staff, were involved at all study sites. A detailed overview of authorship contributions for each country can be found in each respective article.
